# Hydatid Cyst of the Pancreas: An Unusual Cause of Abdominal Pain

**DOI:** 10.7759/cureus.20614

**Published:** 2021-12-22

**Authors:** Yasmine Cherouaqi, Anass Nadi, Anass Idrissi, Abdennaceur El Idrissi Lamghari, Fedoua Rouibaa

**Affiliations:** 1 Gastroenterology and Proctology, Faculty of Medicine, Mohammed VI University of Health Sciences (UM6SS) Cheich Khalifa International University Hospital, Casablanca, MAR; 2 Visceral Surgery, Faculty of Medicine, Mohammed VI University of Health Sciences (UM6SS) Cheich Khalifa International University Hospital, Casablanca, MAR

**Keywords:** surgical treatment, diagnosis, pancreatic cystic lesion, pancreas, hydatid cyst, pancreatic hydatid cyst

## Abstract

Pancreas is an uncommon site of hydatid cysts (HCs) even in endemic countries. Primary pancreatic hydatid cysts (PHCs) mainly occur through hematogenous dissemination. Their rarity and the absence of clinical manifestations in most cases explain their challenging preoperative diagnosis. In symptomatic cases, clinical findings may be similar to those of other diseases. We report a case of a 54-year-old female presented with a six-month history of abdominal pain, although her abdominal examination was normal. Radiological imaging revealed a serous cyst in the body and tail of the pancreas. Biliopancreatic endoscopic ultrasound (EUS) suggested a peritoneal hydatid cyst. Intraoperatively, it was diagnosed as a PHC. The patient underwent resection of the PHC and was then placed on albendazole. She did not have any symptoms for the last seven months. Through this case report, we can conclude that peritoneal hydatid cyst of the pancreas should be considered in the differential diagnosis of the cystic lesions of the pancreas. Moreover, surgery achieves a definitive treatment of the disease.

## Introduction

Hydatid cyst (HC) is a disease caused by a parasite called *Echinococcus granulosus*. It is an endemic zoonosis in the Mediterranean area [1]. It often occurs in the liver and lung; however, the primary pancreatic localization of HC is rare. Primary pancreatic hydatid cysts (PHCs) are often diagnosed incidentally [2]. They can be difficult to differentiate from cysts or pseudocysts of the pancreas. The mainstay of therapy for a PHC is the radical resection of the PHC and albendazole administration. Surgical procedures depend on the location of the HC in the pancreas [3]. In this case report, we present the case of a PHC as an unusual cause of abdominal pain that was misdiagnosed as a peritoneal HC on endoscopic ultrasound (EUS). We also present a review to increase the knowledge on the diagnosis and management of PHCs.

## Case presentation

**Figure 1:**
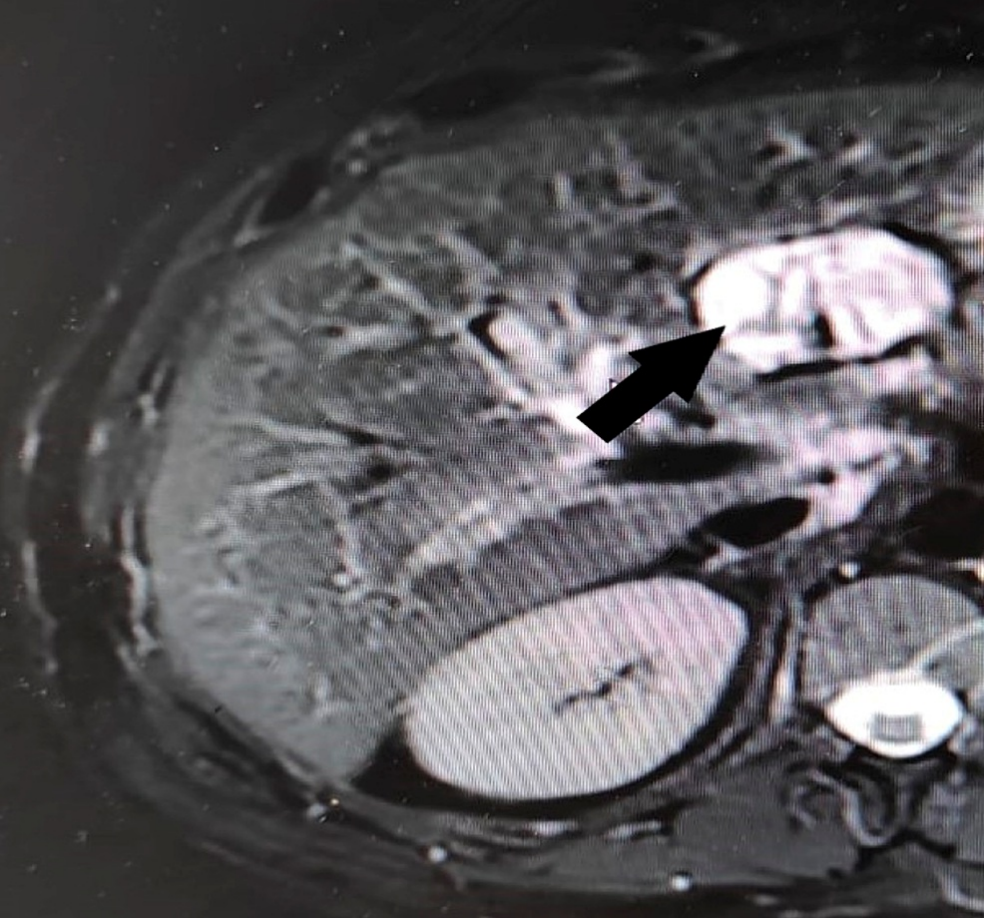
Abdominal MRI showing a serous cyst located in the isthmus of the pancreas

We present a case of a 54-year-old female living in Morocco, who has a past medical history of hypothyroidism on levothyroxine sodium. She presented with a six-month history of abdominal pain in the epigastrium and left hypochondrium region, with no other digestive or extradigestive symptoms. She had no history of pancreatitis, anorexia, or weight loss. Abdominal examination and the biology report were normal. Abdominal ultrasonography (USG) was suspicious for a cystic mass of the pancreas. Abdominal magnetic resonance imaging (MRI) showed a serous cyst located in the isthmus of the pancreas measuring 48 × 36 mm (Figure 1).

**Figure 2:**
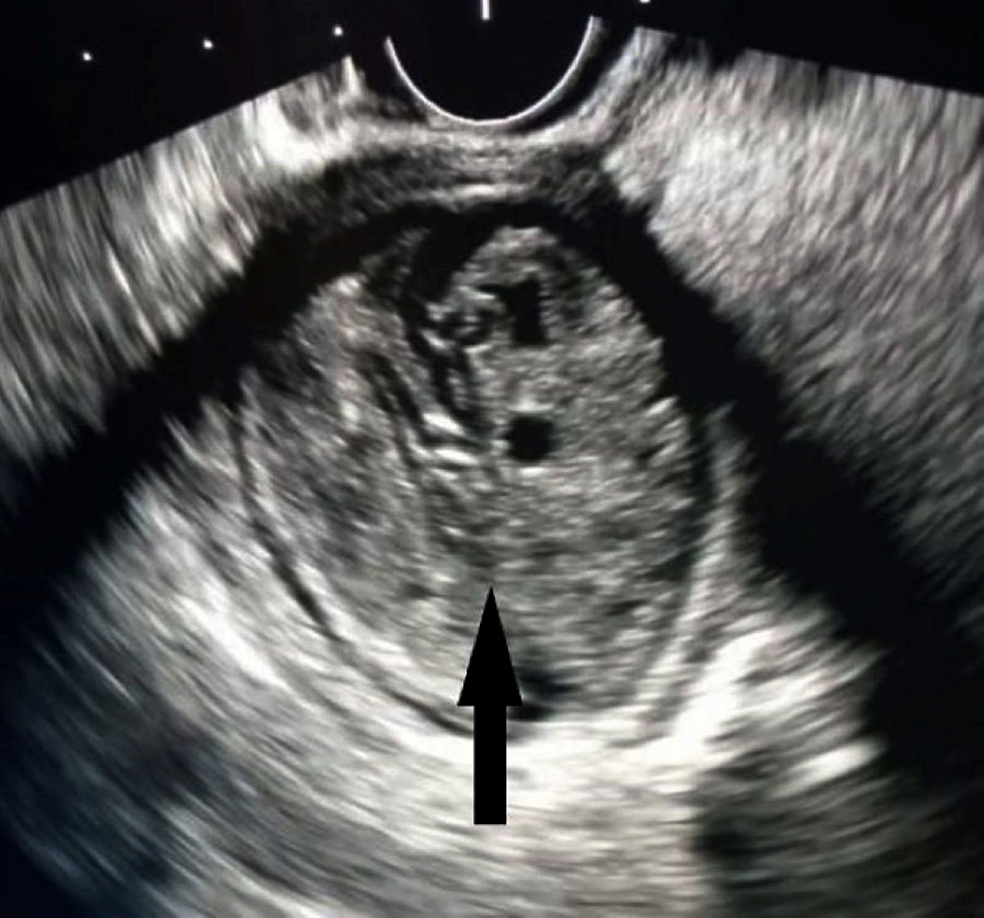
Aspect of hydatid cyst of the pancreas on EUS

**Figure 3:**
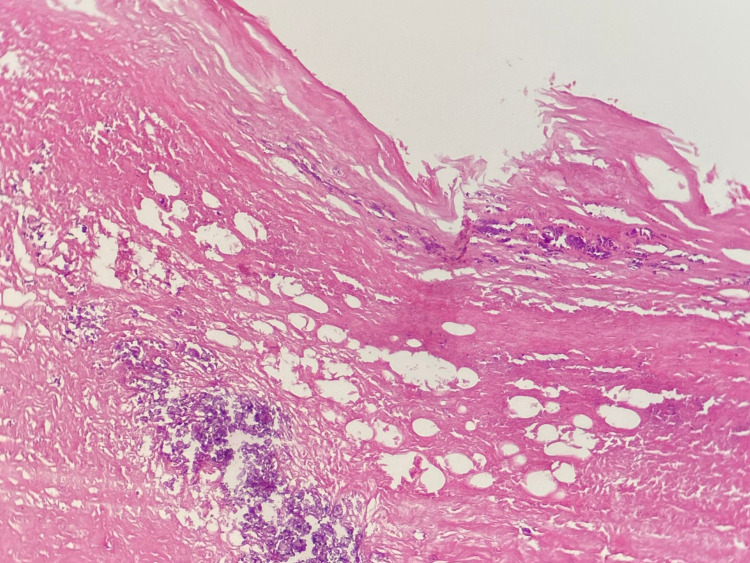
The densely fibrous wall of hydatid cyst traversed calque reshuffle (hematoxylin and eosin stain)

The biliopancreatic EUS showed a heterogeneous extrapancreatic mass, it was oval, hyperechoic, and measured 49.5 × 38 mm. It had an echogenic double wall and contained serpiginous elements evoking hydatid membranes, suggesting a peritoneal HC (Figure 2). Hydatid serology was negative and the final diagnosis was made intraoperatively. The surgical exploration of the abdominal cavity led to the discovery of a type IV PHC, according to the Gharbi classification. In the major axis, it measured 50 mm. Treatment consisted of resection of the protruding dome of the PHC, and the postoperative period was uneventful. The pathology report confirmed the diagnosis of PHC (Figure 3). The patient underwent antiechinococcosis treatment; she was placed on albendazole 800 mg/day, for three months, three courses each 21 days, with one week gap between the courses. Postoperatively, she remained symptom-free for the last seven months. Repeat abdominal USG did not detect any hydatid recurrence.

## Discussion

PHCs are a rare cause of cystic lesions of the pancreas [4]. They are rare, with an incidence rate ranging from 0.14% to 2% [5]. They are exceptionally reported in children [6]. Pancreatic cyst grows slowly at a rate of 0.3-2 cm/year [7], and their size is variable from few millimeters to several centimeters [6]. In 90% of cases, PHCs are solitary; they are located in the pancreatic head in 50-58% of the cases [5].

The most accepted mechanism of the pancreatic infestation of hydatid disease is hematogenous dissemination. Other possible mechanisms are the passage of cystic elements to the pancreas through biliary or lymphatic system [4].

PHCs are asymptomatic in most cases; therefore, they are incidentally discovered. Their symptomatology depends on their size, location, and potential complications [7]. They can present with vomiting, weight loss, abdominal mass, and epigastric pain [8]. In the present case report, the patient had abdominal pain in the epigastrium and left hypochondrium region.

The complications of PHC depend on their location in the pancreas. When located in the head of the pancreas, it can cause obstructive jaundice, cholangitis, acute and chronic pancreatitis, duodenal stenosis or fistula, and pancreatic abscess. When located in the pancreatic tail, it can cause splenomegaly, portal hypertension, abscess formation, or it can rupture into the peritoneal cavity and gastrointestinal tract [8].

Abdominal USG is the most sensitive tool for the detection of floating membranes, hydatid sand, and floating daughter cysts. The highly suggestive computed tomography (CT) scanning signs of HC are the water lily sign, water attenuation, and calcifications. EUS can provide a characterization of the cystic lesions of the pancreas, but is limited for making the definitive diagnosis [9]. However, in the present case report, EUS could lead to the diagnostic confirmation of HC, although the diagnosis of the pancreatic location of the HC was made intraoperatively. The fluid aspiration can differentiate malignant cystic lesions [9]. *Echinococcus* antigens in enzyme-linked immunosorbent assay can distinguish PHC from other types of cystic lesions [10]. However, it is not always positive in the presence of HC, as in the present case report, but it is more specific than imagery [9].

The differential diagnoses of PHC are cystic diseases like pseudocyst, serous cystadenoma, and intraductal papillary mucinous neoplasm [11]. In this case report, the PHC was misdiagnosed as a peritoneal HC.

The treatment modalities depend on the location of the PHC. When it is located in the tail of the pancreas, distal pancreatectomy can be a successful treatment option. The treatment option for PHC located in the body and head of the pancreas is proper evacuation, pericystectomy, and omentoplasty. In the case of obstructive jaundice, ultrasound-guided drainage of the HC can be performed. In the case of cholangitis due to PHCs, endoscopic retrograde cholangiopancreatography and mini-sphincterotomy can be an effective treatment strategy [10]. Postoperative medical therapy is albendazole at 800 mg/day or 10-15 mg/kg/day, for three months, three courses each 21 days, with one week gap between the courses. When PHC is diagnosed before surgery, preoperative albendazole is rarely used to decrease the risk of recurrence [12]. In the present case report, the patient underwent surgical treatment and was administered with albendazole.

## Conclusions

PHCs are rare even in endemic areas. They are diagnosed incidentally in most cases. When symptomatic, the clinical manifestation depends on their location in the pancrea, they are an unusual cause of abdominal pain. Despite radiological imaging, including abdominal USG, CT scanning, EUS, and hydatid serology, the preoperative diagnosis of PHC remains difficult. This diagnosis should be considered in the differential diagnosis of cystic lesions of the pancreas, especially in endemic geographical regions. Surgery offers a complete cure of the disease, and the risk of recurrence decreases with the administration of albendazole.
